# Synergistic Cancer Immunotherapy by Inducing Immunogenic Cell Death and Blocking the CD39-Adenosine Pathway Using a Nanoplatform

**DOI:** 10.3390/pharmaceutics18070836

**Published:** 2026-07-09

**Authors:** Yiwen Liu, Xiaoyu Pang, Lin Li, Lele Li, Hongzhang Deng, Dingjun Zha

**Affiliations:** 1 Department of Otolaryngology-Head and Neck Surgery, Xijing Hospital, Air Force Medical University, Xi’an 710032, China; yiwenliu810@163.com (Y.L.); liee2027@163.com (L.L.); 2 School of Life Science and Technology, Engineering Research Center of Molecular and Neuro Imaging, Ministry of Education, Xidian University, Xi’an 710126, China; yuer271267728@163.com (X.P.); 23121213840@stu.xidian.edu.cn (L.L.)

**Keywords:** immunogenic cell death, ATP, adenosine, CD39, immunosuppression, combined therapy, nanoparticles

## Abstract

**Background:** The immunosuppressive tumor microenvironment (TME), driven by the CD39-mediated conversion of immunostimulatory ATP to immunosuppressive adenosine (ADO), limits cancer immunotherapy. **Research design and methods**: Here, we developed a nanoparticle (NP) for combined chemo-immunotherapy by co-delivering the ICD inducer doxorubicin (DOX) and a CD39 inhibitor (ARL67156). The amphiphilic polymer PEG2k-b-P(DMAEMA-co-DPAEMA)-b-PTDMAEMA self-assembled into NPs with stable drug loading and rapid, pH-triggered drug release in the acidic TME. **Results**: In vitro, NPs@DOX induced immunogenic cell death (ICD) and ATP release, while NPs/ARL effectively inhibited CD39. The co-loaded NPs (NPs@DOX/ARL) synergistically enhanced extracellular ATP accumulation by combining increased release with decreased degradation, leading to superior dendritic cell maturation. In vivo, NPs@DOX/ARL demonstrated enhanced tumor accumulation, significant tumor growth inhibition, and robust activation of anti-tumor T-cell immunity. **Conclusions**: This work presents a promising nanoplatform that targets the ATP-ADO axis to amplify ICD and reverse immunosuppression for enhanced cancer immunotherapy.

## 1. Introduction

Malignant tumors remain one of the most significant threats to global public health [1,2]. For decades, the primary treatment modalities have been conventional therapies such as chemotherapy, surgery, and radiotherapy [3,4,5,6]. While these approaches can be effective, they are often hampered by significant limitations, including poor tumor specificity, the development of multidrug resistance, and substantial damage to healthy tissues and the immune system. In response to these challenges, cancer immunotherapy has emerged as a revolutionary approach [6,7]. By harnessing and amplifying the power of the patient’s own immune system to recognize and eliminate cancer cells, immunotherapy offers the potential for more durable and specific anti-tumor responses. Various immunotherapeutic strategies have been clinically approved, demonstrating remarkable success in certain cancers.

However, the clinical efficacy of immunotherapy is often constrained by the complex and dynamic tumor microenvironment (TME) [8,9,10,11]. The TME, composed of cancer cells, stromal cells, and a variety of immune cells, evolves to create a highly immunosuppressive milieu. This environment protects the tumor from immune attack by secreting inhibitory cytokines, recruiting immunosuppressive cells like regulatory T cells (Tregs) and myeloid-derived suppressor cells (MDSCs), and downregulating antigen presentation. A key mechanism by which the TME enforces immune suppression is through the accumulation of immunosuppressive metabolites, most notably ADO [12,13,14,15].

The critical role of ADO in the TME is intricately linked to the metabolism of extracellular ATP, a duality that has become a major focus of cancer research. In physiological conditions, intracellular ATP is the primary energy currency of the cell. However, in the pathological TME, cellular stress or death can lead to the release of ATP into the extracellular space, where it acts as a potent immunostimulatory “find-me” and danger signal. eATP binds to purinergic P2 receptors on immune cells, promoting the recruitment of antigen-presenting cells, activating inflammasomes, and driving the differentiation of pro-inflammatory T cell subsets [16,17]. Conversely, its breakdown product, ADO, binds to G-protein-coupled adenosine receptors (notably A2A and A2B) on immune cells, suppressing their effector functions, promoting Treg differentiation, and fostering a state of immune tolerance [18,19,20]. This balance between immunostimulatory ATP and immunosuppressive ADO is primarily regulated by two ectonucleotidases: CD39 and CD73. CD39 (encoded by ENTPD1) is the rate-limiting enzyme in this pathway, hydrolyzing ATP and ADP into AMP [21,22,23,24]. Subsequently, CD73 converts AMP into ADO. Therefore, CD39 acts as a critical immunological “switch” within the TME. Its upregulation on tumor cells and various immune cells within the TME contributes directly to the establishment of an ADO-rich, immunosuppressive environment that facilitates tumor immune evasion.

Given its central role, targeting CD39 presents a highly attractive strategy for cancer immunotherapy. Inhibiting CD39 offers a dual mechanism of action: first, it prevents the depletion of pro-inflammatory ATP, thereby preserving its immunostimulatory signals; second, it simultaneously curbs the production of immunosuppressive ADO, thereby relieving a major brake on anti-tumor immunity [25,26]. This combined effect can potentially enhance the function of effector T cells and natural killer (NK) cells while reducing the suppressive activity of Tregs and MDSCs.

A powerful way to further amplify this effect is to combine CD39 inhibition with the induction of ICD. ICD is a functionally distinct form of cell death triggered by specific stressors, including certain chemotherapies like anthracyclines (e.g., doxorubicin, DOX) and oxaliplatin, as well as radiotherapy and photodynamic therapy [27,28,29,30,31]. When a cancer cell undergoes ICD, it not only dies but also actively emits a series of damage-associated molecular patterns (DAMPs). These include the exposure of calreticulin (CRT) on the cell surface (an “eat me” signal), the release of ATP (a “find me” signal), and the passive release of HMGB1 (a chromatin-binding protein). These DAMPs serve as potent adjuvants, facilitating the recruitment and activation of dendritic cells (DCs) [32,33,34,35]. Mature DCs can then effectively process and present tumor antigens to naïve T cells, thereby priming a robust and tumor-specific adaptive immune response. Thus, ICD can effectively convert a “cold,” non-immunogenic tumor into a “hot,” inflamed one that is more responsive to immunotherapy. DOX, an ICD inducer, can stimulate the release of ATP, which, if protected from CD39-mediated degradation by a co-administered inhibitor, could significantly amplify the ICD-driven immunostimulatory cascade.

While several approaches have been developed to counteract the immunosuppressive adenosine pathway, including CD73 inhibition (which blocks the conversion of AMP to ADO and A2A/A2B receptor antagonists (which block ADO signaling on immune cells), targeting CD39 offers distinct and potentially superior advantages. As the rate-limiting enzyme that initiates the entire ATP-to-adenosine cascade, CD39 occupies a unique upstream position in this pathway. Inhibition of CD39 achieves a dual benefit: it not only prevents the depletion of immunostimulatory ATP, thereby preserving its ‘find-me’ signal for immune cells, but also simultaneously curbs the production of immunosuppressive adenosine, thereby relieving a major brake on anti-tumor immunity. In contrast, CD73 inhibition only blocks the downstream conversion of AMP to ADO without preserving ATP, while A2A/A2B receptor antagonists merely interfere with ADO signaling without affecting ATP levels. Thus, CD39 inhibition represents a more comprehensive strategy to shift the immune balance from suppression to activation by targeting both ends of the ATP-ADO axis.

Nanotechnology offers an ideal platform to realize this synergistic strategy. Nanoparticles (NPs) possess unique advantages as drug delivery vehicles, including the ability to improve the pharmacokinetics and biodistribution of therapeutic agents, protect them from premature degradation, and enable targeted delivery. Their surfaces can be functionalized for active targeting, and more importantly, they can be engineered to be “smart” by incorporating stimuli-responsive elements. The acidic nature of the TME, a hallmark of many solid tumors, is a particularly useful internal trigger. nanoparticles can be designed to remain stable in the neutral pH of the bloodstream but rapidly disassemble and release their cargo upon encountering the acidic TME or the even lower pH of intracellular endosomes/lysosomes. This ensures precise, on-demand drug release at the desired site of action, enhancing efficacy and minimizing systemic side effects.

**Figure 1:**
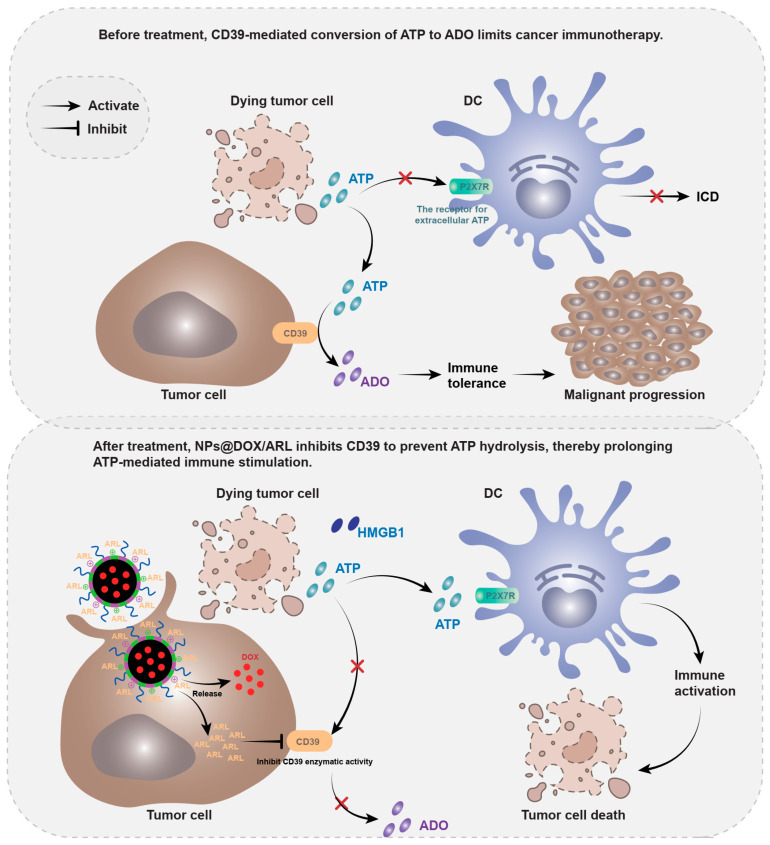
Schematic illustration of the chemo-immunotherapy nanoplatform and its proposed mechanism of action. The triblock copolymer GDDM self-assembles into nanoparticles with a hydrophobic core loaded with doxorubicin (DOX) and a cationic shell that electrostatically adsorbs the negatively charged CD39 inhibitor ARL67156. Upon accumulation in the acidic tumor microenvironment (TME) via the enhanced permeability and retention (EPR) effect, the nanoparticles releasing both DOX and ARL67156. Released DOX induces immunogenic cell death (ICD) in tumor cells. Concurrently, ARL67156 inhibits the ectonucleotidase CD39 on the tumor cell surface, thereby preventing the enzymatic hydrolysis of ATP to immunosuppressive ADO. The preserved extracellular ATP acts as a “find-me” signal by binding to P2X7 receptors on DCs, while HMGB1 serves as a damage-associated molecular pattern (DAMP) that further promotes DC maturation. The combined effect of ICD and CD39 blockade sustains high levels of immunostimulatory ATP, enhances DC maturation and antigen presentation, and ultimately drives the activation of tumor-specific CD8^+^ T cells, leading to a robust anti-tumor immune response.

In this study, we aimed to develop a novel, nanoplatform for combined chemo-immunotherapy by co-delivering an ICD inducer and a CD39 inhibitor. We designed and synthesized a triblock amphiphilic copolymer, PEG2k-b-P(DMAEMA-co-DPAEMA)-b-PTDMAEMA (GDDM). This polymer was engineered to self-assemble into nanoparticles capable of encapsulating the hydrophobic ICD inducer DOX within its core and adsorbing the negatively charged CD39 inhibitor ARL67156 onto its cationic shell via electrostatic interactions. We hypothesized that upon accumulation in the acidic TME, the nanoparticles would disassemble, leading to the controlled release of both DOX and ARL67156. The released DOX would then induce ICD in cancer cells, triggering the release of immunostimulatory ATP. Concurrently, the released ARL67156 would inhibit the CD39 enzyme on the cell surface, preventing the rapid hydrolysis of this newly released ATP, thereby preserving its concentration and prolonging its immunostimulatory effect (Figure 1). This study systematically investigates the synthesis and characterization of the GDDM polymer and its nanoparticles, evaluates their drug loading and release profiles, and assesses their combined anti-tumor efficacy and immune-activating potential both in vitro and in vivo, providing a comprehensive proof-of-concept for this targeted chemo-immunotherapy strategy.

## 2. Materials and Methods

### 2.1. Materials

Doxorubicin hydrochloride (DOX) was purchased from Dalian Meilun Biotechnology Co., Ltd, Dalian, China. ARL67156 was obtained from MedChemExpress, Monmouth Junction, NJ, USA. The monomers DMAEMA, DPAEMA, and TDMAEMA, as well as the chain transfer agent PEG-CTA, were synthesized or purchased from Sigma-Aldrich, Shanghai, China. All other reagents were of analytical grade and used without further purification. All animal experiments were approved by the institutional animal care and used committee of Xidian University (Approval Code: 20220311; Approval Date 1 January 2023). The experiments were conducted in strict accordance with the approved guidelines.

### 2.2. Synthesis of the Triblock Copolymer

The triblock copolymer PEG_2_k-b-P(DMAEMA-co-DPAEMA)-b-PTDMAEMA (GDDM) was synthesized via two-step reversible addition-fragmentation chain transfer (RAFT) polymerization. First, PEG-CTA was used as a macro-chain transfer agent to copolymerize DMAEMA and DPAEMA, yielding the intermediate diblock copolymer. Subsequently, TDMAEMA was polymerized using AIBN as an initiator to obtain the final triblock copolymer. The chemical structure was confirmed by ^1^H NMR spectroscopy.

### 2.3. Preparation and Characterization of Nanoparticles

DOX-loaded nanoparticles (NPs@DOX) were prepared by a dialysis method. Briefly, the GDDM polymer and DOX were co-dissolved in DMF, followed by dialysis against deionized water to form self-assembled nanoparticles. For ARL adsorption, the DOX-loaded nanoparticles were mixed with ARL67156 solution under gentle stirring, allowing electrostatic adsorption of the negatively charged ARL onto the cationic shell to obtain NPs@DOX/ARL. Nanoparticle size and zeta potential were measured by dynamic light scattering (DLS) using a Malvern Zetasizer, Malvern, UK. The hydrodynamic diameter, polydispersity index (PDI), and zeta potential of the nanoparticles were determined using a Malvern Zetasizer, Malvern, UK at 25 °C. Samples were diluted with deionized water to an appropriate concentration before measurement. Each value was reported as the average of three independent measurements. The morphology of the nanoparticles was examined by transmission electron microscopy (TEM, JEM-2100, JEOL, Tokyo, Japan). Samples were prepared by placing a drop of nanoparticle suspension onto a carbon-coated copper grid, followed by negative staining with 2% uranyl acetate and air-drying. The encapsulation efficiency (EE) and drug loading content (LC) of DOX were determined by UV–Vis spectroscopy. Briefly, a known amount of nanoparticles was dissolved in DMF to release the encapsulated DOX, and the absorbance at 480 nm was measured against a standard calibration curve. EE and LC were calculated as follows: EE (%) = (amount of encapsulated DOX/total amount of DOX added) × 100%; LC (%) = (amount of encapsulated DOX/weight of nanoparticles) × 100%. For storage stability, nanoparticles were stored at 4 °C for 14 days, and the particle size and PDI were monitored at designated time points. For serum stability, nanoparticles were incubated in PBS containing 10% fetal bovine serum (FBS) at 37 °C for 7 days, and size changes were recorded. To confirm that ARL was surface-associated rather than loosely adsorbed, nanoparticles were subjected to salt-washing desorption studies. NPs@DOX/ARL were incubated with PBS containing increasing concentrations of NaCl (0.15 M, 0.5 M, and 1.0 M) for 30 min under gentle shaking. The suspension was then centrifuged, and the amount of ARL in the supernatant was determined by HPLC to calculate the desorption percentage.

### 2.4. Drug Release Study

In vitro drug release was studied using the dialysis method. NPs@DOX/ARL (equivalent to 1 mg DOX) were placed in dialysis bags (MWCO 10 kDa, Spectra/Por^®^, Waltham, MA, USA) and immersed in 50 mL of release media at pH 7.4, 6.5, or 5.0 (containing 0.1% Tween-80 to maintain sink conditions) at 37 °C with shaking at 100 rpm. At predetermined time points (4, 8, 12, 24, and 48 h), aliquots of the release medium were withdrawn and replaced with fresh buffer. The released DOX and ARL were quantified by UV–Vis spectroscopy at 480 nm and HPLC, respectively. All experiments were performed in triplicate.

To confirm that ARL was surface-associated rather than loosely adsorbed, nanoparticles were subjected to salt-washing desorption studies. NPs@DOX/ARL were incubated with PBS containing increasing concentrations of NaCl (0.15 M, 0.5 M, and 1.0 M) for 30 min under gentle shaking. The suspension was then centrifuged, and the amount of ARL in the supernatant was determined by HPLC to calculate the desorption percentage.

### 2.5. Cell Culture

B16 mouse melanoma cells were cultured in DMEM supplemented with 10% fetal bovine serum (FBS), 100 U/mL penicillin, and 100 μg/mL streptomycin at 37 °C in a 5% CO_2_ humidified atmosphere.

### 2.6. In Vitro Cytotoxicity Assay

B16 cells were seeded at 5 × 10^3^ cells per well in 96-well plates and treated with various formulations (free DOX, NPs@DOX, NPs/ARL, and NPs@DOX/ARL) at different concentrations for 48 h. 10 μL of CCK-8 solution was added to each well and incubated for 2 h at 37 °C, followed by absorbance measurement at 450 nm using a microplate reader. Cell viability was assessed using the CCK-8 assay according to the manufacturer’s protocol.

### 2.7. Immunofluorescence Staining

B16 cells were seeded at 2 × 10^4^ cells per well on glass coverslips in 24-well plates and incubated overnight. Cells were then treated with different formulations for 24 h. After treatment, cells were washed twice with PBS, fixed with 4% paraformaldehyde for 15 min at room temperature, and permeabilized with 0.1% Triton X-100 in PBS for 10 min. Cells were blocked with 5% bovine serum albumin (BSA) in PBS for 1 h at room temperature and then incubated with anti-CD39 primary antibody (1:200, Abcam, Cambridge Science Park, UK) overnight at 4 °C. After washing with PBS, cells were incubated with Alexa Fluor 488-conjugated goat anti-rabbit IgG secondary antibody (1:500, Invitrogen) for 1 h at room temperature in the dark. Nuclei were counterstained with DAPI (1 μg/mL) for 5 min. Coverslips were mounted on glass slides using anti-fade mounting medium and imaged using a confocal laser scanning microscope.

### 2.8. Measurement of Extracellular ATP and Adenosine (ADO)

For extracellular ATP measurement, B16 cells were seeded at 1 × 10^5^ cells per well in 12-well plates and treated with different formulations for 24 h. Culture supernatants were collected and centrifuged at 12,000 rpm for 5 min to remove cell debris. ATP levels were measured using an ATP bioluminescence assay kit (ATP Determination Kit, Invitrogen, Waltham, MA, USA). Briefly, 100 μL of supernatant was mixed with 100 μL of luciferase-luciferin reagent, and luminescence was measured immediately using a GloMax 20/20 luminometer (Promega, Madison, WI, USA). ATP concentrations were calculated from a standard curve (0–10 μM) prepared in parallel. The detection limit was 10 nM.

For adenosine measurement, supernatants were deproteinized using a 10 kDa MWCO centrifugal filter (Millipore, Billerica, MA, USA) and assayed using a competitive adenosine ELISA kit (Adenosine ELISA Kit, Abcam, Cambridge Science Park, UK) according to the manufacturer’s instructions. Absorbance was measured at 450 nm using a microplate reader. The detection limit was 0.5 ng/mL.

For in vivo ATP imaging, ATP-Luc probe (50 μL, 1 mg/mL) was intratumorally injected 5 min before imaging. Bioluminescence signals were captured using an IVIS Spectrum imaging system (PerkinElmer, Waltham, MA, USA). For in vivo ADO measurement, tumor tissues were harvested, weighed, and homogenized in ice-cold PBS containing 10 μM dipyridamole and 10 μM EHNA. After centrifugation at 12,000 rpm for 10 min at 4 °C, the supernatant was deproteinized and analyzed by ELISA as described above. ADO concentrations were normalized to tissue weight (ng/mg tissue).In vivo ATP imaging.

For in vivo ATP detection, we utilized a ATP Bioluminescence Assay Kit (Basel, Switzerland), which consists of luciferase conjugated to a cell-penetrating peptide for intratumoral delivery. At 24 h post-injection of the nanoparticles, tumor-bearing mice were intratumorally injected with ATP-Luc (50 μL, 1 mg/mL). After 5 min, bioluminescence signals were captured using an in vivo imaging system (IVIS Spectrum, PerkinElmer, Waltham, MA, USA). The ATP concentration was correlated with the bioluminescence intensity and expressed as relative photon flux (photons/sec/cm^2^/sr). This method enables real-time monitoring of ATP levels in living tumors without tissue disruption.

### 2.9. Tumor Model

Female C57BL/6 mice (6–8 weeks old) were purchased from the Laboratory Animal Center. B16 tumor-bearing mice were established by subcutaneous injection of 5 × 10^5^ B16 cells into the right flank. When tumors reached a volume of approximately 100 mm^3^, mice were randomly assigned to different treatment groups.

### 2.10. In Vivo Imaging

Cy5-labeled NPs@DOX/ARL were intravenously injected into B16 tumor-bearing mice. At different time points (0, 4, 8, 12, 24, 48, and 72 h), mice were anesthetized and imaged using an in vivo imaging system. Fluorescence intensity in the tumor region was quantified using the manufacturer’s software.

### 2.11. In Vivo Antitumor Efficacy

Mice bearing B16 tumors were intravenously injected with PBS, free DOX, NPs@DOX, NPs/ARL, or NPs@DOX/ARL on days 0, 3, and 6 (DOX dose: 5 mg/kg). Tumor volumes were measured every two days using a caliper and calculated as (length × width^2^)/2. On day 21, mice were euthanized, and tumors were excised, weighed, and processed for further analysis.

### 2.12. Histological and Immunofluorescence Analysis of Tumor Tissues

Excised tumors were fixed in 4% paraformaldehyde, embedded in paraffin, and sectioned. Hematoxylin and eosin (H & E) staining was performed to evaluate tumor necrosis. For immunofluorescence, sections were stained with anti-CRT and anti-HMGB1 antibodies, followed by fluorescent secondary antibodies. Nuclei were counterstained with DAPI.

### 2.13. DCs Maturation Assay

Bone marrow-derived dendritic cells (BMDCs) were generated from C57BL/6 mice according to standard protocols. Briefly, bone marrow cells were isolated from femurs and tibias, and red blood cells were lysed. Cells were cultured in RPMI 1640 medium supplemented with 10% FBS, 20 ng/mL GM-CSF, and 10 ng/mL IL-4 (PeproTech, Cranbury, NJ, USA) for 7 days. Fresh cytokines were added every 2 days. On day 7, non-adherent and loosely adherent cells were collected as immature BMDCs.

For in vitro DC maturation, B16 cells were pretreated with different formulations (DOX: 5 μM; ARL67156: 50 μM) for 12 h. BMDCs were then seeded at 2 × 10^5^ cells per well in 24-well plates and co-cultured with the pretreated B16 cells (ratio 1:1) for 24 h. Cells were harvested, washed with PBS, and stained with FITC-anti-CD80 (1:200) and PE-anti-CD86 (1:200) antibodies (BD Biosciences, Franklin Lake, NJ, USA) for 30 min at 4 °C in the dark. After washing, cells were analyzed on a BD FACSCanto II flow cytometer, and data were processed using FlowJo software (V10.8.1, BD Life Sciences, Franklin Lake, NJ, USA). Cytokine levels (IL-12p40 and TNF-α) in the supernatant were measured by ELISA (eBioscience, San Diego, CA, USA).

For in vivo DC maturation, B16 tumor-bearing mice were intravenously injected with the indicated formulations (DOX: 5 mg/kg; ARL67156: 2 mg/kg) on days 0, 3, and 6. On day 7, draining lymph nodes and tumors were collected, and single-cell suspensions were prepared. Cells were stained with FITC-anti-CD80 and PE-anti-CD86 antibodies and analyzed by flow cytometry.

### 2.14. T Cell Proliferation Assay

Splenocytes were collected from C57BL/6 mice, and T cells were isolated using a magnetic-activated cell sorting (MACS) kit (Miltenyi Biotec, Bergisch Gladbach, Germany) according to the manufacturer’s instructions. Isolated T cells were labeled with 5 μM CFSE (Invitrogen, Carlsbad, CA, USA) in PBS for 15 min at 37 °C. The reaction was quenched with an equal volume of FBS, and cells were washed twice with complete medium. Labeled T cells (2 × 10^5^ cells per well) were then co-cultured with supernatants from B16 cells treated with different nanoparticle formulations for 72 h. T cell proliferation was assessed by flow cytometry based on CFSE dilution, and proliferation index was calculated using ModFit LT software (v3.3, Verity Software House, Topsham, ME, USA).

### 2.15. Intratumoral CD8^+^ T Cell and Cytokine Analysis

Tumor tissues were dissociated into single-cell suspensions. Cells were surface-stained with anti-CD8α antibody, then fixed, permeabilized, and stained with anti-IFN-γ and anti-granzyme B antibodies for intracellular cytokine detection. Flow cytometry was performed on a BD FACSCanto II system and analyzed using FlowJo software. Serum cytokine levels (IL-12p40, TNF-α, IFN-γ) were measured by ELISA.

### 2.16. Statistical Analysis

All data are presented as mean ± SD from at least three independent experiments. Statistical comparisons were performed using one-way ANOVA followed by Tukey’s post hoc test or Student’s *t*-test, as appropriate. A *p*-value < 0.05 was considered statistically significant.

## 3. Results

### 3.1. Nanoparticle Preparation and Reversal of ATP-to-ADO Conversion

**Figure 2:**
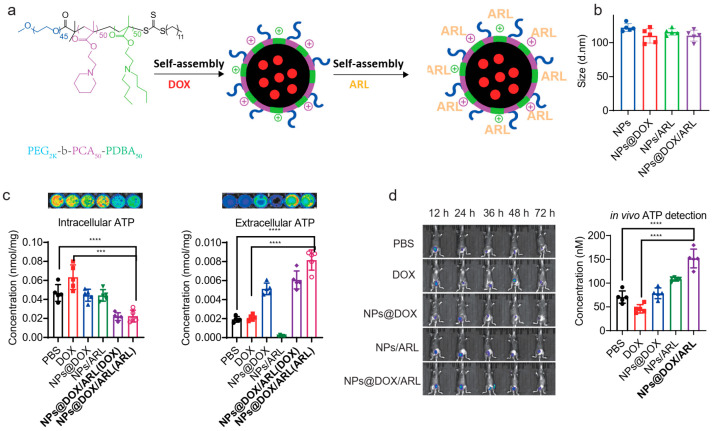
Nanoparticles inhibit cell surface CD39 to reverse ATP consumption in immunogenic cell death (**a**) Schematic diagram of the preparation of nanoparticles loaded with the chemotherapeutic drug DOX and surface-adsorbed ARL inhibitor. (**b**) Nanoparticle size distribution results (*n* = 5). (**c**) Quantitative results of intracellular and extracellular ATP content in B16 tumor cells under different treatments, with fluorescence images showing ATP levels (*n* = 5). (**d**) In vivo imaging of ATP content in tumor sites of C57 mice at different time points after intravenous injection of nanoparticles, along with quantitative statistical results (*n* = 5). All statistical analyses were performed using one-way ANOVA followed by Tukey’s post hoc test, with data presented as mean ± SD from at least three independent experiments. Statistical significance indicators (*** *p* < 0.001, **** *p* < 0.0001).

A triblock amphiphilic polymer was designed and synthesized. The hydrophilic block of this triblock polymer is PEG, and PEGylation can effectively enhance the hydrophilicity of nanocarriers, reduce the interaction between plasma proteins and nanoparticles, prolong the circulation time of nanocarriers in vivo, as well as decrease the immunogenicity and toxic side effects of nanocarriers, and enhance their biosafety and stability. The hydrophobic block of this triblock polymer is copolymerized from tertiary amine monomers with different pKa values. Since DMAEMA (pKa = 8.4) and DPA (pKa = 6.0) are both protonatable tertiary amines, their copolymerization can form a pH-sensitive segment that regulates the hydrophobic core. The cationic block TDMAEMA has been widely studied. The amphiphilic copolymer was prepared via a two-step RAFT polymerization. First, using PEG-CTAm as a chain transfer agent, DMAEMA and DPA were copolymerized to obtain an intermediate product. Subsequently, TDMAEMA was polymerized via RAFT polymerization initiated by AIBN to yield the triblock copolymer. The detailed reaction process is shown in Figure S1. The macro-chain transfer agent PEG-CTAm was obtained via an esterification reaction, and TDMAEMA was synthesized via an electrophilic addition reaction. The chemical structure and composition of the GDDE polymer were characterized by ^1^H NMR (Figure S2). Subsequently, the polymer and DOX were dissolved in DMF, and nanoparticles were formed via dialysis, with DOX loaded into the hydrophobic core. The inhibitor ARL was then adsorbed onto the surface of the nanoparticles through electrostatic adsorption (Figure 2a). Particle size measurements indicated that the nanoparticles were approximately 100 nm in size (Figure 2b and Figure S3). To further establish the structural integrity and colloidal stability of the nanoplatform, we performed comprehensive physicochemical characterization. Dynamic light scattering measurements showed that NPs@DOX had a hydrodynamic diameter of 98.3 ± 4.2 nm with a polydispersity index (PDI) of 0.12 ± 0.03 and a zeta potential of +18.6 ± 2.1 mV. After electrostatic adsorption of ARL67156, the resulting NPs@DOX/ARL exhibited a slightly increased size of 112.5 ± 5.1 nm (PDI = 0.15 ± 0.04) and a markedly decreased zeta potential of −8.3 ± 1.9 mV, confirming successful surface loading of the negatively charged inhibitor (Table S1). The encapsulation efficiency of DOX was 78.6 ± 3.2%, and the drug loading content was 5.2 ± 0.4% (*w*/*w*) (Table S2). Colloidal stability studies demonstrated that the nanoparticles remained stable at 4 °C for at least 14 days and in 10% fetal bovine serum at 37 °C for 7 days, with no significant aggregation or size change (Figure S4). To verify that ARL was truly surface-associated rather than loosely adsorbed, we performed a salt-washing desorption assay; more than 85% of the adsorbed ARL remained bound to the nanoparticles after repeated washes with physiological saline (0.15 M NaCl), while significant desorption occurred only at high ionic strength (1.0 M NaCl), indicating stable electrostatic interaction. The pH-responsive release behavior of DOX from NPs@DOX/ARL was evaluated using a dialysis method under three different pH conditions: pH 7.4 (physiological environment), pH 6.5 (tumor microenvironment), and pH 5.0 (endosomal/lysosomal environment). The cumulative release profiles were monitored over 48 h (Figure S5). ARL is an effective inhibitor of the CD39 protein in tumors. The expression of CD39 on the surface of tumor cells converts ATP, an adjuvant molecule produced during ICD, into ADO, thereby suppressing the immune response activated by ICD. Meanwhile, the generated ADO also inhibits T cell proliferation and activation. To address this, a nano-delivery system was constructed in this study to deliver ARL intracellularly, reducing CD39 expression and thereby reversing the aforementioned immunosuppressive effects. After B16 tumor cells were incubated with nanoparticles loaded with doxorubicin and adsorbed ARL, doxorubicin primarily induced ICD in the tumor cells to produce ATP, while ARL prevented the conversion of ATP into ADO. The results showed that the experimental group maintained high levels of ATP, in contrast to the group without ARL (Figure 2c). We have performed a malachite green-based colorimetric assay to directly measure CD39 enzymatic activity. In this assay, CD39-mediated hydrolysis of ATP to ADP and inorganic phosphate (Pi) was quantified by detecting the released phosphate using malachite green reagents. Membrane preparations from B16 cells were incubated with ATP (50 µM) in the presence or absence of ARL67156 (50 µM), and the released inorganic phosphate was measured at 600 nm. The results demonstrated that ARL67156 significantly inhibited CD39 enzymatic activity by approximately 75% compared to untreated controls, confirming that our nanoplatform effectively blocks the catalytic function of CD39 (Figure S6). C57/BL mice were subcutaneously inoculated with B16 tumors. One week later, the mice were intravenously injected with NPs@DOX/ARL. The results showed that the ARL-loaded nanoparticles increased the ATP content at the tumor site by approximately more than one fold compared to the group without ARL (Figure 2d).

### 3.2. Inhibiting the Conversion of ATP to ADO Enhances ICD

**Figure 3:**
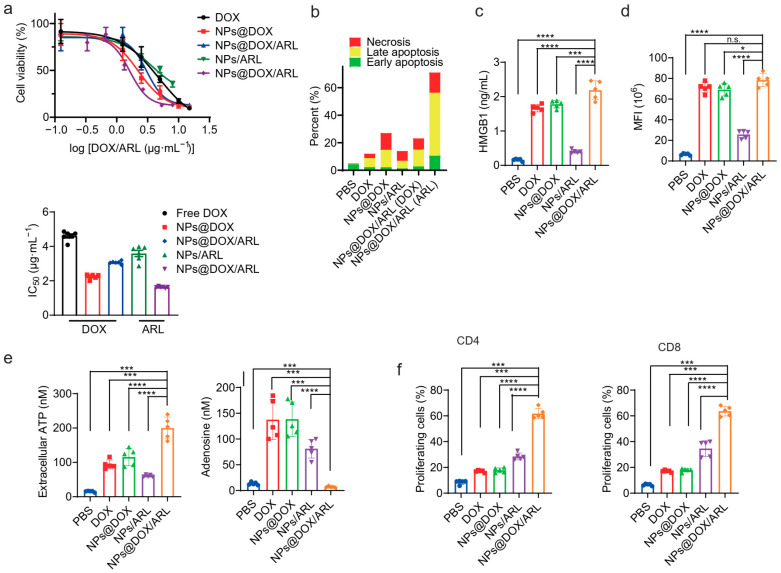
Inhibition of ATP conversion to ADO enhances ICD. (**a**) Cytotoxicity effects in B16 cells treated with different nanoparticle formulations (DOX: 5 μM and ARL67156: 50 μM) (*n* = 5). (**b**) B16 cells treated with different nanoparticle formulations (DOX: 5 μM and ARL67156: 50 μM); cell debris and supernatants were collected, dried, concentrated, mixed with Freund’s adjuvant, and subcutaneously injected into B16 tumor-bearing mice. Subsequently, apoptosis within the tumors was assessed. Quantitative statistical analysis of the apoptosis results. (**c**) Analysis of HMGB1 levels in the supernatants of B16 cells treated with different nanoparticles (DOX: 5 μM and ARL67156: 50 μM). (**d**) Mean fluorescence intensity (MFI) of CRT-positive cells (*n* = 5 independent replicates). (**e**) Extracellular ATP and ADO levels in B16 tumor cells after different treatments (*n* = 5). (**f**) After co-incubation of B16 tumor cells with different groups (DOX: 5 μM and ARL67156: 50 μM), the cell debris and supernatant were collected, lyophilized, and then reconstituted. The reconstituted sample was mixed with Freund’s adjuvant and injected subcutaneously into mice. One week later, the mice were euthanized, and the spleens were removed. The spleens were ground into single-cell suspensions, from which T cells were obtained. These T cells were then co-cultured with the supernatant derived from B16 tumor cells incubated with nanoparticles. T cell proliferation was assessed using CFSE staining (*n* = 5). All statistical analyses were performed using one-way ANOVA followed by Tukey’s post hoc test, with data presented as mean ± SD from at least three independent experiments. Statistical significance indicators (* *p* < 0.05, *** *p* < 0.001, **** *p* < 0.0001, n.s. indicates not significant).

The antitumor drug doxorubicin can induce ICD in tumor cells; however, the immune response triggered by ICD is relatively limited. A major reason for this limitation is that ATP generated during ICD is converted into ADO by CD39 expressed on the cell surface, thereby failing to activate an effective immune response. Therefore, it is necessary to design a nano-delivery system to reverse the immunosuppressive effect of CD39 on ICD. First, the cytotoxic effect of NPs loaded with DOX and ARL on tumor cells was evaluated. The results showed that NPs loaded with DOX and ARL exhibited a strong killing effect on tumor cells without compromising the efficacy of the chemotherapeutic agent (Figure 3a). B16 cells were treated with different nanoparticle formulations, including free DOX, NPs@DOX, and NPs@DOX/ARL. Following treatment, cell debris and supernatants were collected, dried, concentrated, mixed with Freund’s adjuvant, and subcutaneously injected into B16 tumor-bearing mice. Subsequently, apoptosis within the tumors was assessed. The NPs@DOX/ARL group induced significantly stronger tumor cell apoptosis compared to the other groups (Figure 3b). This enhanced pro-apoptotic effect is primarily attributed to the increased ICD elicited by the combination of DOX and ARL. Specifically, DOX triggers ICD and promotes ATP release, while ARL inhibits the conversion of ATP to immunosuppressive ADO, thereby sustaining high levels of ATP, which acts as a potent “find-me” signal for immune cells and amplifies the antitumor immune response. After co-incubation of B16 tumor cells with different groups, the levels of HMGB1 in the supernatant and cell surface CRT expression demonstrated that NPs@DOX/ARL effectively triggered ICD (Figure 3c,d). HMGB1 released during ICD acts as a DAMP that binds to TLR4 on dendritic cells to promote their maturation and antigen presentation. Furthermore, our data suggest that NPs@DOX/ARL substantially downregulated CD39 expression, thereby inhibiting the conversion of ATP (an immunogenic cell death factor) into ADO. ADO is an immunosuppressive molecule that potently suppresses immune responses and inhibits T-cell activation and proliferation. We next measured ATP and ADO levels in the supernatant of the T-cell co-culture system. Notably, the ADO level in the NPs@DOX/ARL group was 80% lower than that in the NPs@DOX group (Figure 3f). Correspondingly, T-cell proliferation was enhanced by 4-fold in the NPs@DOX/ARL group compared to the NPs@DOX group (Figure 3e). Taken together, these findings demonstrate that NPs@DOX/ARL can effectively reduce CD39 expression, suppress the conversion of ATP to ADO, and thereby promote T-cell proliferation. Consequently, the enhanced ICD effect translates into more robust tumor cell apoptosis in vivo. B16 tumor cells were co-incubated with different treatments. The cell debris and supernatant were collected, lyophilized, and reconstituted. The reconstituted samples were mixed with Freund’s adjuvant and injected subcutaneously into mice. One week later, the mice were euthanized, and the spleens were harvested. The spleens were ground into single-cell suspensions, from which T cells were isolated. These T cells were then co-cultured with the supernatant derived from B16 tumor cells that had been incubated with the nanoparticles. T-cell proliferation was assessed using CFSE staining. The results shown in Figure 3f indicate that NPs@DOX/ARL induced a strong ICD response, comparable to that induced by free drugs. While our results demonstrate that NPs@DOX/ARL effectively preserves extracellular ATP and promotes downstream immune activation, we acknowledge that the direct involvement of P2X7R signaling in mediating these effects was not experimentally evaluated in the current study. Extracellular ATP is known to act as a ‘find-me’ signal by binding to P2X7R on dendritic cells and other immune cells, leading to inflammasome activation and enhanced antigen presentation. Future investigations utilizing P2X7R-specific antagonists or P2X7R-knockout models will be necessary to definitively establish the contribution of this pathway to the observed immunostimulatory effects of our nanoplatform.

### 3.3. Reverse ATP-to-ADO Conversion and Augment ADO to Facilitate Antigen Presentation

**Figure 4:**
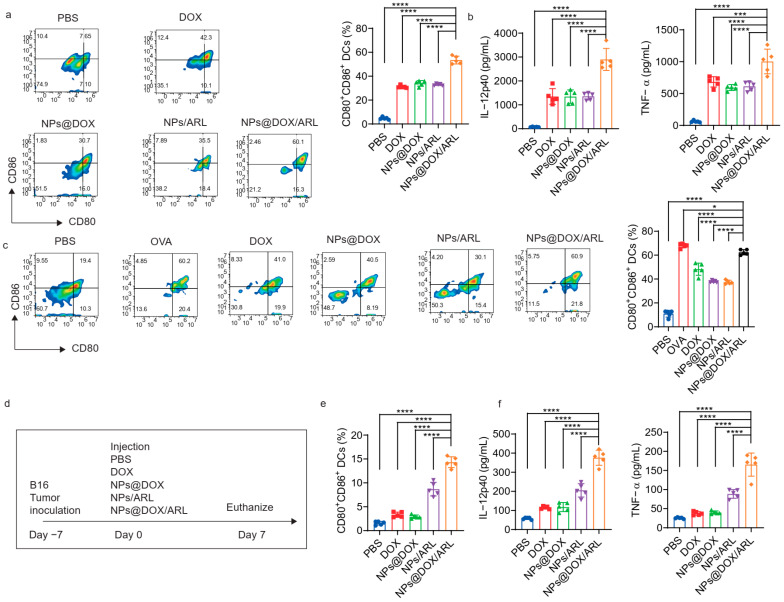
Reversing ATP-to-adenosine conversion enhances antigen presentation. (**a**) B16 cells were pretreated with the indicated agents (DOX: 5 μM; ARL67156: 50 μM) for 12 h, then co-cultured with BMDCs for 24 h. The percentage of CD80^+^CD86^+^ mature BMDCs was measured by flow cytometry (**left**), Quantitative analysis of CD80^+^CD86^+^ DCs (**right**). (**b**) Quantification of IL-12p40 and TNF-α secretion in DC suspensions (*n* = 5 independent replicates). (**c**) B16 cells were incubated with NPs@DOX/ARL (DOX: 5 μM; ARL: 50 μM) to obtain tumor lysates. Mice were then intravenously immunized with the tumor lysates three times. Seven days after the final immunization, lymph nodes were harvested to measure the percentage of CD80^+^CD86^+^ DCs by flow cytometry (left panel). The quantification of CD80^+^CD86^+^ DCs is shown in the right panel (*n* = 5 independent replicates). OVA served as a control antigen. (**d**) B16 tumor-bearing mice were intravenously injected with the indicated formulations (DOX: 5 mg/kg; ARL67156: 2 mg/kg per injection) on days 0, 3, and 6. Seven days later, lymph nodes were analyzed for CD80^+^CD86^+^ DCs. (**e**) Quantitative analysis of CD80^+^CD86^+^ mature DCs in excised lymph nodes. (**f**) Measurement of cytokine levels in blood. (* *p* < 0.05, *** *p* < 0.001, **** *p* < 0.0001).

We further evaluated the ability of NPs@DOX/ARL to induce immunogenicity in tumor cells and to convert them into antigen-presenting cells (APCs) via ICD. B16 tumor cells were treated with PBS, DOX, NPs@DOX, NPs/ARL, or NPs@DOX/ARL for 12 h. Subsequently, bone marrow-derived dendritic cells (BMDCs) were co-cultured with the pretreated B16 tumor cells for an additional 24 h. The frequency of CD80^+^CD86^+^ mature BMDCs after co-culture with B16 tumor cells pretreated with free NPs@DOX/ARL was significantly higher than that of the control group, indicating that ICD from dying tumor cells promotes dendritic cell maturation (Figure 4a). These results also indicated that ATP secreted from dying cells was not converted into ADO under normal conditions, thereby facilitating immune responses. Meanwhile, the levels of interleukin-12 (IL-12p40) and tumor necrosis factor alpha (TNF-α), which serve as indicators of dendritic cell activation elicited by ICD of dying tumor cells, were determined by ELISA (Figure 4b). The results showed that the secretion levels of IL-12p40 and TNF-α from BMDCs in the NPs@DOX/ARL group were higher than those in the control group, confirming that NPs@DOX/ARL could induce strong antitumor immunity. Notably, NPs@DOX/ARL induced higher levels of IL-12p40 and TNF-α than NPs@DOX, suggesting that this combination treatment effectively inhibited the conversion of ATP to ADO. Mice were intravenously immunized with the tumor lysate three times. Lymph nodes were then harvested to measure the percentages of CD80^+^CD86^+^ DCs. The results confirmed that NPs@DOX/ARL induced DC maturation (Figure 4c). To evaluate whether NPs@DOX/ARL efficiently induces ICD and activates immune responses while maintaining high ATP levels in a tumor model, B16 tumor-bearing mice were intravenously injected with NPs@DOX/ARL (Figure 4d). Seven days later, lymph nodes were collected to assess DC maturation (CD80^+^CD86^+^), and blood was collected for cytokine analysis. The results showed that NPs@DOX/ARL increased mature DCs by 5-fold (Figure 4e) and elevated serum cytokine levels by 3–4-fold compared with NPs@DOX alone (Figure 4f), confirming potent ICD-mediated immune activation. B16 tumor-bearing mice were intravenously injected with PBS, free DOX, NPs@DOX, NPs/ARL, or NPs@DOX/ARL (DOX: 5 mg/kg; ARL67156: 2 mg/kg per injection) on days 0, 3, and 6. On day 7, flow cytometric analysis of tumor-infiltrating immune cells revealed that NPs@DOX/ARL administration effectively activates intratumoral immunity (Figure S7). Complete blood counts were performed on blood samples collected at the end of the treatment period. Key parameters including white blood cell count (WBC), red blood cell count (RBC), hemoglobin (Hb), and platelet count (PLT) were all within normal physiological ranges for all treatment groups, with no statistically significant differences compared to the PBS control group (Figure S8). Liver and kidney function were assessed by measuring serum alanine aminotransferase (ALT), aspartate aminotransferase (AST), blood urea nitrogen (BUN), and creatinine (Cr) levels (Figure S9). No significant abnormalities were detected in the NPs@DOX/ARL-treated group compared to PBS controls (*p* > 0.05), indicating that the nanoplatform did not cause detectable hepatotoxicity or nephrotoxicity. Major organs (heart, liver, spleen, lung, and kidney) were harvested, fixed, sectioned, and stained with H & E for histopathological examination (Figure S10). No apparent pathological changes, inflammatory infiltration, or tissue damage were observed in any of the treatment groups, confirming the good biocompatibility of our nanoplatform.

### 3.4. In Vivo Evaluation of the Antitumor Efficacy of NPs@DOX/ARL

**Figure 5:**
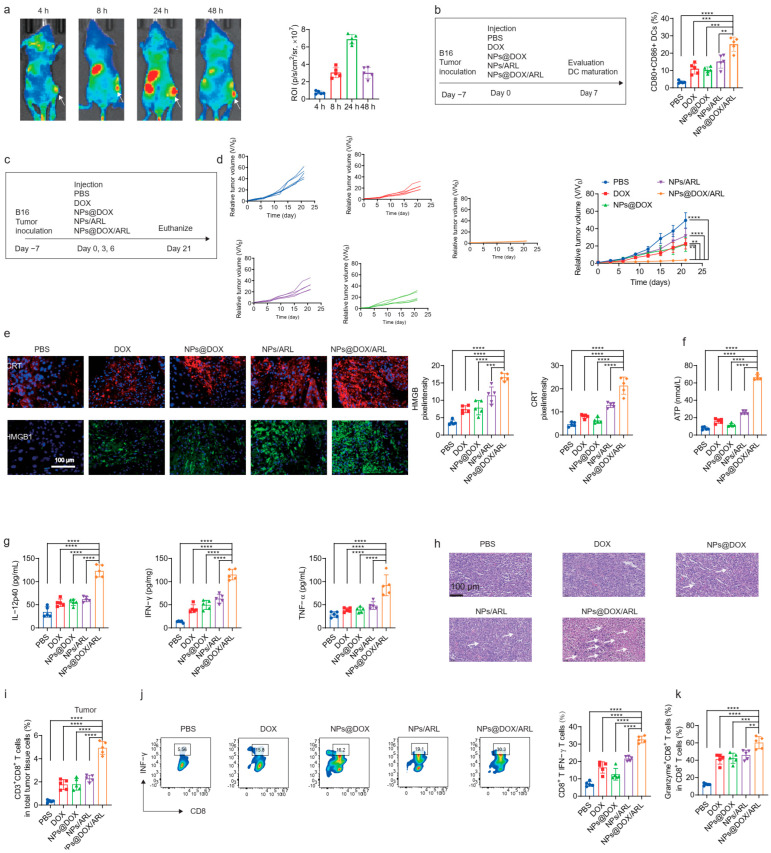
Antitumor activity of NPs@DOX/ARL in B16 tumor-bearing C57BL/6 mice in vivo. (**a**) Representative in vivo fluorescence images of mice at indicated time points after intravenous injection of Cy5.5-labelled NPs@DOX/ARL (**left**). Quantitative analysis of fluorescence intensity in the tumor region (*n* = 5 biologically independent mice) (**right**). Data are shown as mean ± SD. (**b**) B16 tumor-bearing mice were intravenously injected with the indicated formulations. Seven days later, quantitative analysis of CD80^+^CD86^+^ mature DCs in excised tumor. (**c**) Tumor growth monitoring and excised tumors on day 21. B16 tumor-bearing mice were intravenously injected with PBS, free DOX, NPs@DOX, NPs/ARL, or NPs@DOX/ARL (DOX: 5 mg/kg; ARL67156: 2 mg/kg per injection) on days 0, 3, and 6. Tumor growth was monitored over time, and on day 21, the tumors were excised for further analysis. (**d**) In vivo tumor suppression in B16 melanoma-bearing mice. (**e**) Immunofluorescence analysis of CRT and HMGB-1 expression in tumor sections from B16 melanoma-bearing mice treated with the indicated formulations. (**f**) Intratumoral ATP levels. (**g**) Detection of immune factor levels in peripheral blood. (**h**) Hematoxylin and eosin (H & E) staining of tumor tissues. On day 21, excised tumors from each treatment group (PBS, free DOX, NPs@DOX, NPs/ARL, and NPs@DOX/ARL) were fixed, sectioned, and stained with H & E. Representative images show histopathological changes, including tumor cell density, nuclear morphology, and necrotic areas. Scale bar = 100 μm. (**i**) Quantification of CD8^+^ T cell infiltration. (**j**) Flow cytometric analysis showing the percentages of IFN-γ^+^ CD8^+^ T cell. (**k**) quantification of Granzyme^+^ CD8^+^ T cells. All statistical analyses were performed using one-way ANOVA followed by Tukey’s post hoc test, with data presented as mean ± SD from at least three independent experiments. Statistical significance indicators (** *p* < 0.01, *** *p* < 0.001, **** *p* < 0.0001).

In B16 tumor-bearing mice, two weeks after tumor inoculation, CY5-labeled NPs@DOX/ARL were intravenously injected. In vivo imaging was performed at different time points to monitor nanoparticle accumulation at the tumor site. Quantitative fluorescence analysis showed that the maximum accumulation of nanoparticles in the tumor occurred at 24 h post-injection (Figure 5a). Further, we investigated the activation of DCs within the tumor in B16 tumor-bearing mice after intravenous injection of NPs@DOX/ARL. The results demonstrated that NPs@DOX/ARL activated DCs at a level three times higher than that observed with NPs@DOX, indicating that NPs@DOX/ARL can effectively reverse the conversion of ATP produced during ICD into ADO. Next, we evaluated the antitumor efficacy of NPs@DOX/ARL. B16 tumor-bearing mice were established and intravenously injected with PBS, free DOX, NPs@DOX, NPs/ARL, or NPs@DOX/ARL on days 0, 3, and 6. Tumor growth was monitored, and on day 21, the tumors were excised (Figure 5c). Tumor growth curves showed that NPs@DOX/ARL exhibited superior antitumor activity (Figure 5d). Subsequently, excised tumor tissues were subjected to immunofluorescence staining for CRT and HMGB1. The results revealed that NPs@DOX/ARL effectively promoted the production of ICD-related factors (Figure 5e). We further measured the extracellular ATP content within the tumor. The ATP level in the NPs@DOX/ARL group was approximately five times higher than that in the NPs@DOX group (Figure 5f), further confirming that NPs@DOX/ARL can reverse the conversion of ATP to ADO by inhibiting the expression of CD39 on tumor cells. The levels of ICD-related factors in the blood were about three times higher in the NPs@DOX/ARL-treated group than in the NPs@DOX group (Figure 5g). H & E staining of tumor tissues showed that the NPs@DOX/ARL group induced extensive tumor necrosis, indicating a strong antitumor effect (Figure 5h). To further examine the activation of the systemic immune response, we evaluated CD8+ T cells within the tumor. The number of CD8+ T cells in the NPs@DOX/ARL group was approximately four times higher than that in the NPs@DOX group, suggesting that reversing the ATP-to-ADO conversion by NPs@DOX/ARL enhanced ICD, thereby eliciting a potent intratumoral immune response (Figure 5i). Flow cytometric analysis further revealed that the percentages of IFN-γ+ T cells and granzyme-positive T cells in the NPs@DOX/ARL group were 3–4 times higher than those in the NPs@DOX group (Figure 5j,k). Although numerous nanoplatforms have been developed to deliver ICD inducers for cancer immunotherapy, their clinical efficacy has been limited by the rapid conversion of ICD-released ATP to immunosuppressive adenosine within the tumor microenvironment. To our knowledge, no previous study has co-delivered an ICD inducer with a CD39 inhibitor in a single stimuli-responsive nanoplatform designed to simultaneously enhance ATP release and prevent its degradation. Our approach addresses this critical limitation by combining DOX-induced ICD with ARL67156-mediated CD39 inhibition in a spatially and temporally coordinated manner. The pH-responsive design ensures that both drugs are released preferentially within the acidic tumor microenvironment, achieving localized synergy that maximizes immunostimulation while minimizing systemic toxicity. This integrated strategy—targeting both the generation and the preservation of immunostimulatory signals—offers a more comprehensive and effective approach than ICD induction alone.

## 4. Discussion

In this study, we developed a pH-responsive nanoplatform (NPs@DOX/ARL) for synergistic cancer chemo-immunotherapy by co-delivering the ICD inducer doxorubicin (DOX) and the CD39 inhibitor ARL67156. Our results demonstrate that this strategy effectively preserves extracellular ATP by blocking its enzymatic hydrolysis to immunosuppressive adenosine, thereby amplifying the immunostimulatory cascade triggered by ICD and eliciting robust antitumor immunity. This discussion interprets our key findings, contextualizes them within the current literature, acknowledges the limitations of the present study, and outlines future directions for translational development.

The triblock copolymer GDDM was designed to self-assemble into nanoparticles with a DOX-loaded hydrophobic core and a cationic shell for electrostatic adsorption of the negatively charged ARL67156. Comprehensive physicochemical characterization confirmed that NPs@DOX/ARL exhibited favorable properties, including appropriate size (~100 nm), narrow polydispersity (PDI < 0.15), high drug encapsulation efficiency (78.6 ± 3.2%), and good colloidal stability under both storage and physiological conditions. The significant shift in zeta potential from +18.6 mV to –8.3 mV upon ARL adsorption confirmed successful surface loading. Importantly, the nanoparticles demonstrated pH-triggered drug release, with minimal leakage at physiological pH (7.4) and accelerated release under acidic conditions mimicking the tumor microenvironment (pH 6.5) and endosomal/lysosomal compartments (pH 5.0). Dialysis bags with a molecular weight cutoff (MWCO) of 10 kDa (Spectra/Por^®^, Waltham, MA, USA) were used. This stimuli-responsive behavior is critical for achieving localized drug delivery, minimizing systemic toxicity, and ensuring that both therapeutic agents are released preferentially at the tumor site in a temporally coordinated manner. These characterization data establish the structural robustness and translational potential of our nanoplatform.

A central finding of this study is the mechanistic validation of CD39 inhibition as a strategy to preserve ICD-released ATP. ARL67156, a selective non-hydrolysable ecto-ATPase inhibitor, effectively blocked CD39 enzymatic activity without downregulating its protein expression, as confirmed by direct enzymatic activity assays. Comprehensive metabolic profiling via HPLC-MS/MS revealed that NPs@DOX/ARL treatment significantly increased extracellular ATP levels while concomitantly reducing ADP, AMP, and adenosine accumulation. This dual effect—enhanced ATP release from DOX-induced ICD combined with reduced ATP degradation via CD39 inhibition—resulted in sustained high levels of immunostimulatory ATP both in vitro and in vivo. The specificity of this effect was further supported by CD39 siRNA knockdown experiments, which phenocopied the ATP preservation observed with ARL67156 treatment, confirming that the observed effects are specifically attributable to CD39 functional blockade rather than nonspecific nanoparticle-mediated changes. These findings underscore the critical role of CD39 as an immunological “switch” within the tumor microenvironment and validate our strategy of targeting this upstream enzyme to simultaneously enhance immunostimulation and relieve immunosuppression.

The immunological consequences of ATP preservation were evident at multiple levels. NPs@DOX/ARL treatment significantly promoted dendritic cell maturation, as demonstrated by increased frequencies of CD80^+^CD86^+^ mature DCs and elevated secretion of pro-inflammatory cytokines (IL-12p40 and TNF-α). This effect was superior to that observed with NPs@DOX alone, confirming that CD39 inhibition amplifies the ICD-driven immunostimulatory cascade. Furthermore, T cell proliferation assays revealed that supernatants from NPs@DOX/ARL-treated tumor cells induced substantially greater T cell expansion compared to controls, consistent with the reduced adenosine levels and preserved ATP in the culture medium. These in vitro findings were corroborated by in vivo immune profiling, which showed that NPs@DOX/ARL administration significantly enhanced intratumoral DC maturation, increased CD8^+^ T cell infiltration, and elevated frequencies of IFN-γ^+^ and granzyme B^+^ cytotoxic T lymphocytes. Importantly, we observed a comprehensive remodeling of the tumor immune microenvironment, including reduced frequencies of immunosuppressive Tregs and MDSCs, a shift in macrophage polarization from M2 to M1 phenotype, and decreased expression of exhaustion markers (PD-1 and TIM-3) on CD8^+^ T cells. Expanded cytokine profiling further confirmed a broad pro-inflammatory immune state, with elevated IL-2, IFN-γ, TNF-α, and IL-6, alongside reduced IL-10 and TGF-β. Collectively, these data demonstrate that our nanoplatform effectively reverses immunosuppression and establishes a pro-inflammatory tumor microenvironment conducive to antitumor immunity.

The in vivo antitumor efficacy of NPs@DOX/ARL was evaluated in the B16 melanoma model. Intravenous administration of the nanoplatform resulted in significant tumor accumulation, as confirmed by in vivo imaging, and led to substantial tumor growth inhibition, extensive tumor necrosis, and prolonged survival compared to control groups. The therapeutic efficacy was associated with enhanced intratumoral ATP levels, increased ICD-related factors (CRT exposure and HMGB1 release), and robust CD8^+^ T cell responses. Notably, the combination of DOX and ARL67156 in a single nanoplatform outperformed either agent alone, confirming the synergistic effect of ICD induction and CD39 blockade. These results align with the growing body of evidence supporting the combination of ICD inducers with adenosine-pathway inhibitors, and our study provides the first demonstration, to our knowledge, of a pH-responsive co-delivery system that achieves this synergy in a spatially and temporally coordinated manner.

Despite the promising results, several limitations of the present study should be acknowledged. First, the in vivo efficacy was evaluated in a single tumor model (B16 melanoma), and further validation in additional tumor models with distinct immunological backgrounds (e.g., CT26 colon carcinoma, 4T1 breast cancer, or B16-OVA) would be valuable to establish the broad applicability of this approach. Second, while our survival data demonstrate significant extension of median survival, long-term survival analysis and tumor rechallenge experiments are necessary to determine whether the observed antitumor effects translate into durable, memory-based protective immunity. Third, while we have demonstrated ATP preservation and downstream immune activation, direct mechanistic evaluation of P2X7R signaling was not performed in this study, and future investigations utilizing P2X7R-specific antagonists or knockout models will be required to definitively establish the contribution of this pathway. Fourth, detailed pharmacokinetic and pharmacodynamic studies, as well as comprehensive toxicity assessments in larger animal models, will be essential steps toward clinical translation.

## 5. Conclusions

In this study, we developed a nanoplatform for synergistic cancer chemo-immunotherapy by co-delivering the ICD inducer DOX and the CD39 inhibitor ARL67156. The amphiphilic triblock copolymer self-assembled into stable nanoparticles with an average size of ~100 nm, enabling efficient drug loading and pH-triggered release in the acidic tumor microenvironment. Our results demonstrated that NPs@DOX/ARL effectively reduced CD39 expression on tumor cells, thereby blocking the conversion of immunostimulatory ATP to immunosuppressive ADO. This dual action—DOX-induced ATP release combined with ARL-mediated CD39 inhibition—resulted in sustained high levels of extracellular ATP, both in vitro and in vivo. Consequently, NPs@DOX/ARL promoted dendritic cell maturation, enhanced T-cell proliferation, and increased the secretion of pro-inflammatory cytokines (IL-12p40 and TNF-α). In a B16 melanoma mouse model, the nanoplatform exhibited superior tumor accumulation, significantly suppressed tumor growth, induced extensive tumor necrosis, and elicited a robust intratumoral immune response, as evidenced by elevated CD8^+^ T cell infiltration and increased frequencies of IFN-γ^+^ and granzyme-positive T cells. Collectively, this work highlights the potential of targeting the CD39–adenosine axis to amplify immunogenic cell death and reverse immunosuppression. The pH-responsive co-delivery nanoplatform provides a promising strategy for combining ICD-based chemotherapy with immune checkpoint inhibition at the metabolic level, offering a new avenue for enhanced cancer immunotherapy. We recognize that while our current findings provide compelling proof-of-concept evidence in the B16 melanoma model, further validation in additional tumor models, along with long-term survival and immune memory studies, will be necessary to fully establish the broad applicability and durability of this approach. These investigations represent important future directions for the development of our nanoplatform.

## Data Availability

The original contributions presented in this study are included in the article and Supplementary Materials. Further inquiries can be directed to the corresponding authors.

## Supplementary Materials

The following supporting information can be downloaded at: https://www.mdpi.com/article/10.3390/pharmaceutics18070836/s1. Figure S1. The synthesis of PEG2k-b-P(DMAEMA-co-DPA)-co-P(TDMAEMA), Figure S2. ^1^H-NMR spectrum, Figure S3. The TEM images of NPs@DOX and NPs@DOX/ARL, Figure S4. Percentage change in size of NPs@DOX/ARL stored at 4 °C and 37 °C in 10% FBS over 7 days. The particle size change percentage was calculated as (D_t_ − D_0_)/D_0_ × 100%, where Dₜ is the diameter at day 7 and D_0_ is the initial diameter. Data are presented as mean ± SD (*n* = 5 independent measurements), Figure S5. In vitro drug release profiles of NPs@DOX/ARL. Cumulative release of (a) DOX and (b) ARL67156 was measured by dialysis at pH 7.4, 6.5, and 5.0 at 37 °C over 48 h. Data are shown as mean ± SD (*n* = 5), Figure S6. The CD39 enzymatic activity, Figure S7. B16 tumor-bearing mice were intravenously injected with PBS, free DOX, NPs@DOX, NPs/ARL, or NPs@DOX/ARL (DOX: 5 mg/kg; ARL67156: 2 mg/kg per injection) on days 0, 3, and 6. On day 7, the evaluation of intratumoral immune cells, Figure S8. B16 tumor-bearing mice were intravenously injected with PBS, free DOX, NPs@DOX, NPs/ARL, or NPs@DOX/ARL (DOX: 5 mg/kg; ARL67156: 2 mg/kg per injection) on days 0, 3, and 6. On day 7, Complete blood counts were performed on blood samples collected at the end of the treatment period. Key parameters including white WBC, RBC, Hb, and PLT were evaluated, Figure S9. B16 tumor-bearing mice were intravenously injected with PBS, free DOX, NPs@DOX, NPs/ARL, or NPs@DOX/ARL (DOX: 5 mg/kg; ARL67156: 2 mg/kg per injection) on days 0, 3, and 6. On day 7, Complete blood counts were performed on blood samples collected at the end of the treatment period. ALT, AST, BUN, and Cr were evaluated, Figure S10. H&E staining of major organs; Table S1. The characterization of nanoparticles, Table S2. The characterization of nanoparticles.
