# Supplementary material for: Synergistic Cancer Immunotherapy by Inducing Immunogenic Cell Death and Blocking the CD39-Adenosine Pathway Using a Nanoplatform
Source: Pharmaceutics. 2026 Jul 9;18(7):836. doi: 10.3390/pharmaceutics18070836 (PMC13416026; doi:10.3390/pharmaceutics18070836)
Supplement: Supplementary file 1 [file pharmaceutics-18-00836-s001.zip › pharmaceutics-4362280-supplementary.pdf]

Supporting information

Synergistic Cancer Immunotherapy by Inducing Immunogenic Cell Death and Blocking the CD39-Adenosine Pathway Using a Nanoplatform

Yiwen Liu [1], Xiaoyu Pang [2], Lin Li [2], Lele Li [1], Hongzhang Deng [2,*] and Dingjun Zha [1,*]

[1] Department of Otolaryngology-Head and Neck Surgery, Xijing Hospital, Air Force Medical University,
Xi'an 710032, China; yiwenliu810@163.com (Y.L.); liee2027@163.com (L.L.)

[2] School of Life Science and Technology, Engineering Research Center of Molecular and Neuro Imaging, Ministry of Education, Xidian University, Xi'an 710126, China; yuer271267728@163.com (X.P.); 23121213840@stu.xidian.edu.cn (L.L.)

* Correspondence: hzdeng@xidian.edu.cn (H.D.); zhadjun@fmmu.edu.cn (D.Z.)

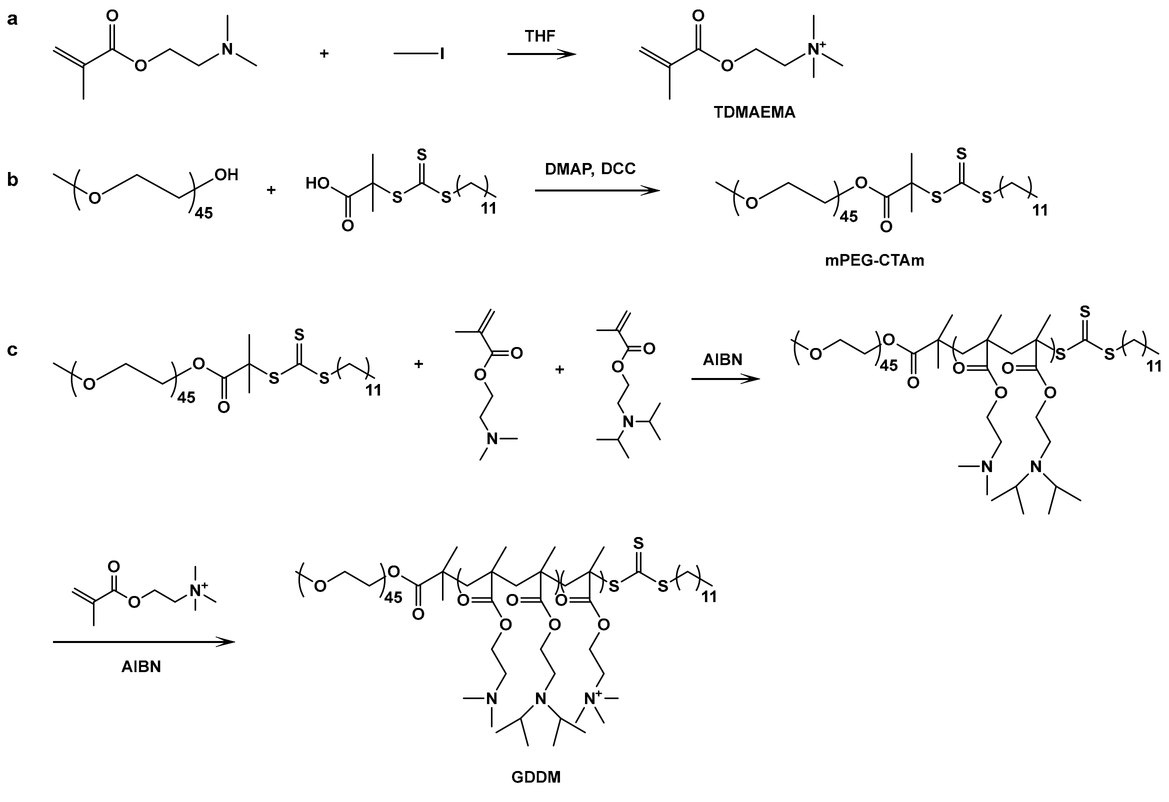

Figure S1. The synthesis of PEG_{2k}-*b*-P(DMAEMA-*co*-DPA)-*co*-P(TDMAEMA)

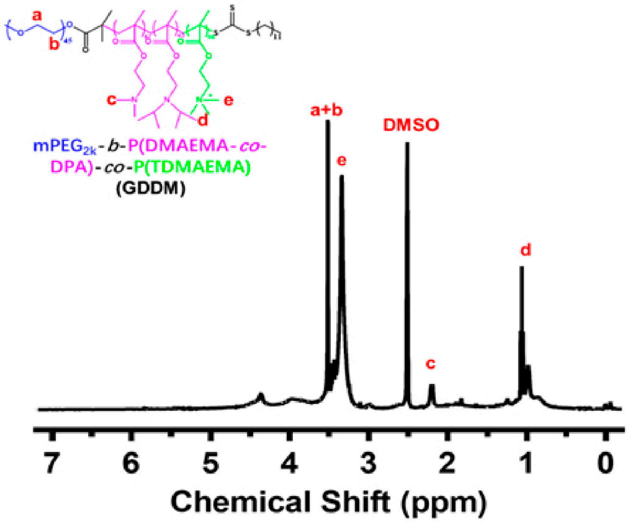

Figure S2. 1H-NMR spectrum.

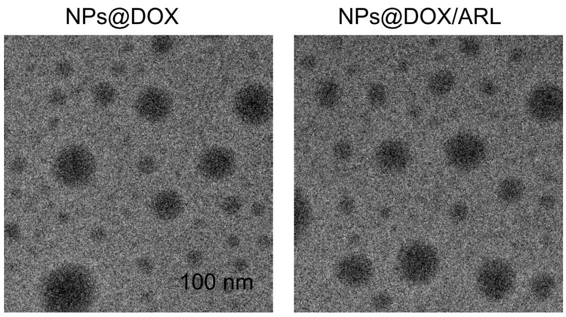

Figure S3. The TEM images of NPs@DOX and NPs@DOX/ARL.

Table S1. The characterization of nanoparticles

	NPs@DOX	NPs@DOX/ARL
Size (nm)	98.3 ± 4.2	112.5 ± 5.1
PDI	0.12 ± 0.03	0.15 ± 0.04
Zeta (mV)	+18.6 ± 2.1	–8.3 ± 1.9

Table S2. The characterization of nanoparticles

	NPs@DOX
Encapsulation efficiency	78.6 ± 3.2%
Drug loading content	5.2 ± 0.4%

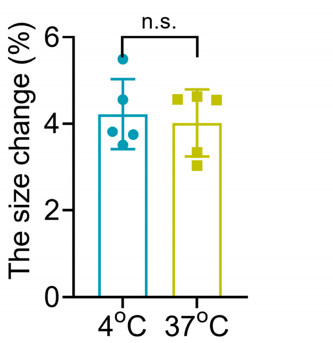

Figure S4. Percentage change in size of NPs@DOX/ARL stored at 4 °C and 37 °C in 10% FBS over 7 days. The particle size change percentage was calculated as $(D_t - D_0)/D_0 \times 100\%$, where D_t is the diameter at day 7 and D_0 is the initial diameter. Data are presented as mean ± SD (n = 5 independent measurements).

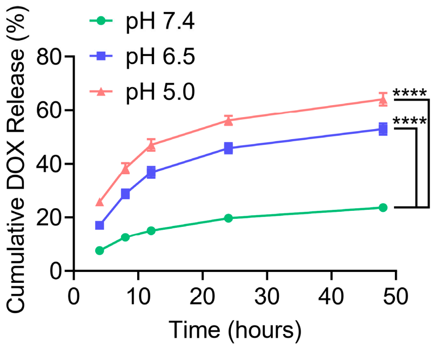

Figure S5. In vitro drug release profiles of NPs@DOX/ARL. Cumulative release of (a) DOX and (b) ARL67156 was measured by dialysis at pH 7.4, 6.5, and 5.0 at 37 °C over 48 h. Data are shown as mean ± SD (n = 5).

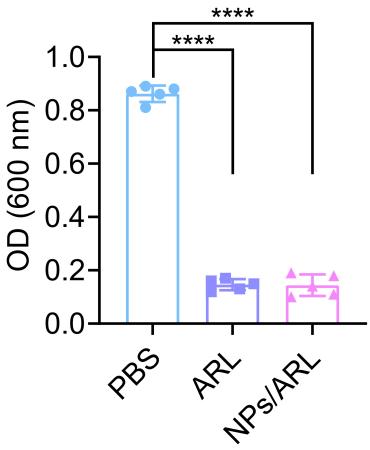

Figure S6. The CD39 enzymatic activity

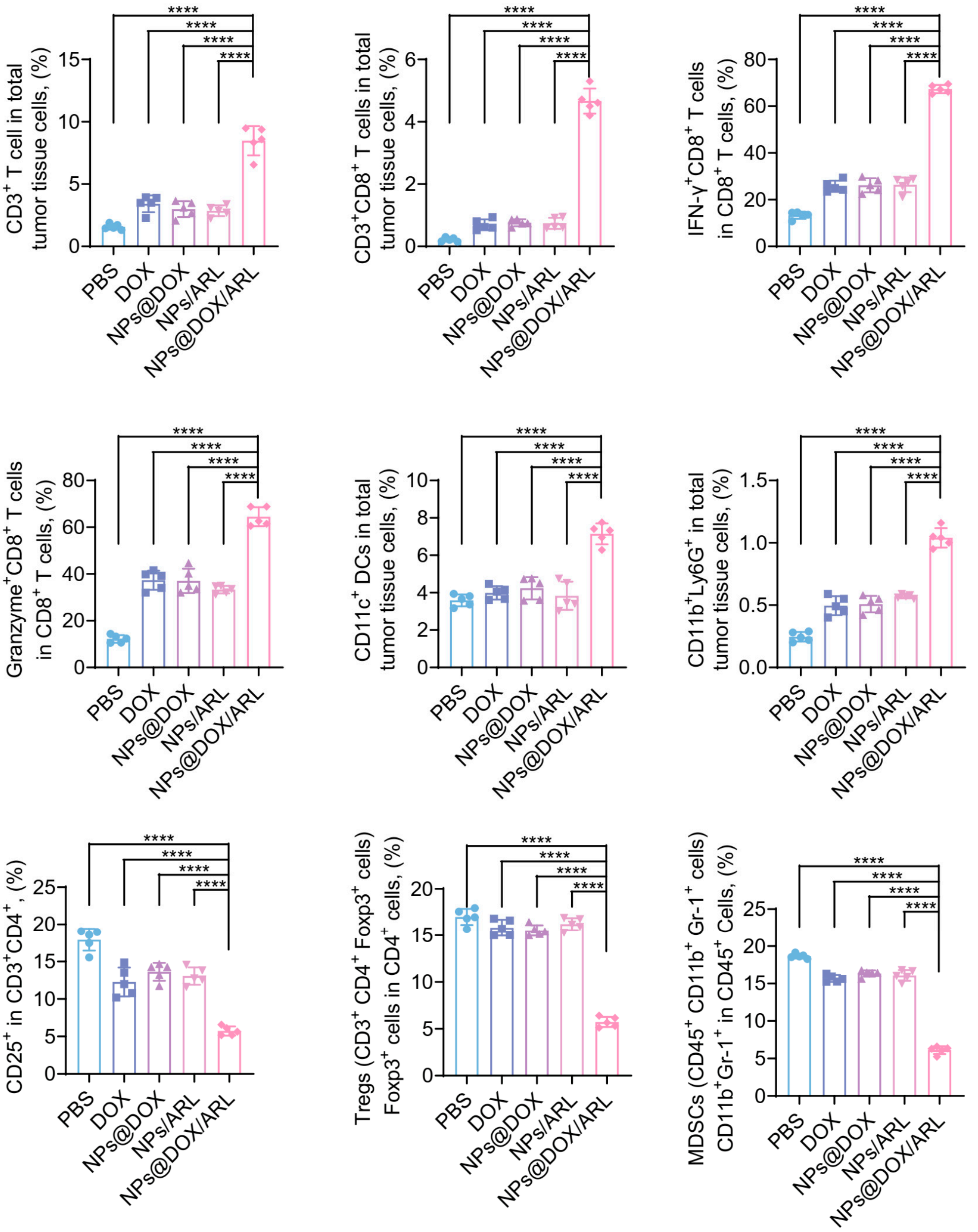

Figure S7. B16 tumor-bearing mice were intravenously injected with PBS, free DOX, NPs@DOX, NPs/ARL, or NPs@DOX/ARL (DOX: 5 mg/kg; ARL67156: 2 mg/kg per injection) on days 0, 3, and 6. On day 7, the evaluation of intratumoral immune cells

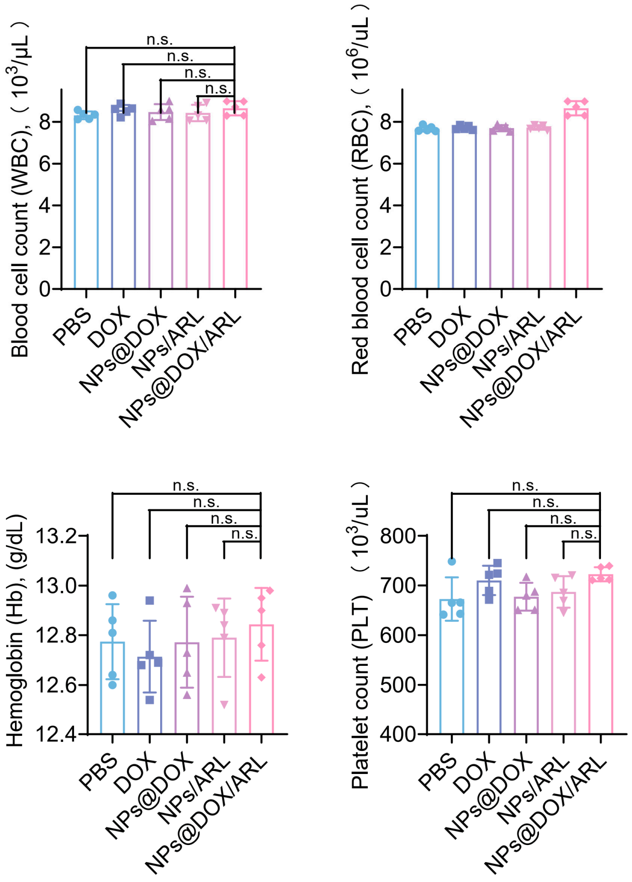

Figure S8. B16 tumor-bearing mice were intravenously injected with PBS, free DOX, NPs@DOX, NPs/ARL, or NPs@DOX/ARL (DOX: 5 mg/kg; ARL67156: 2 mg/kg per injection) on days 0, 3, and 6. On day 7, Complete blood counts were performed on blood samples collected at the end of the treatment period. Key parameters including white WBC, RBC, Hb, and PLT were evaluated.

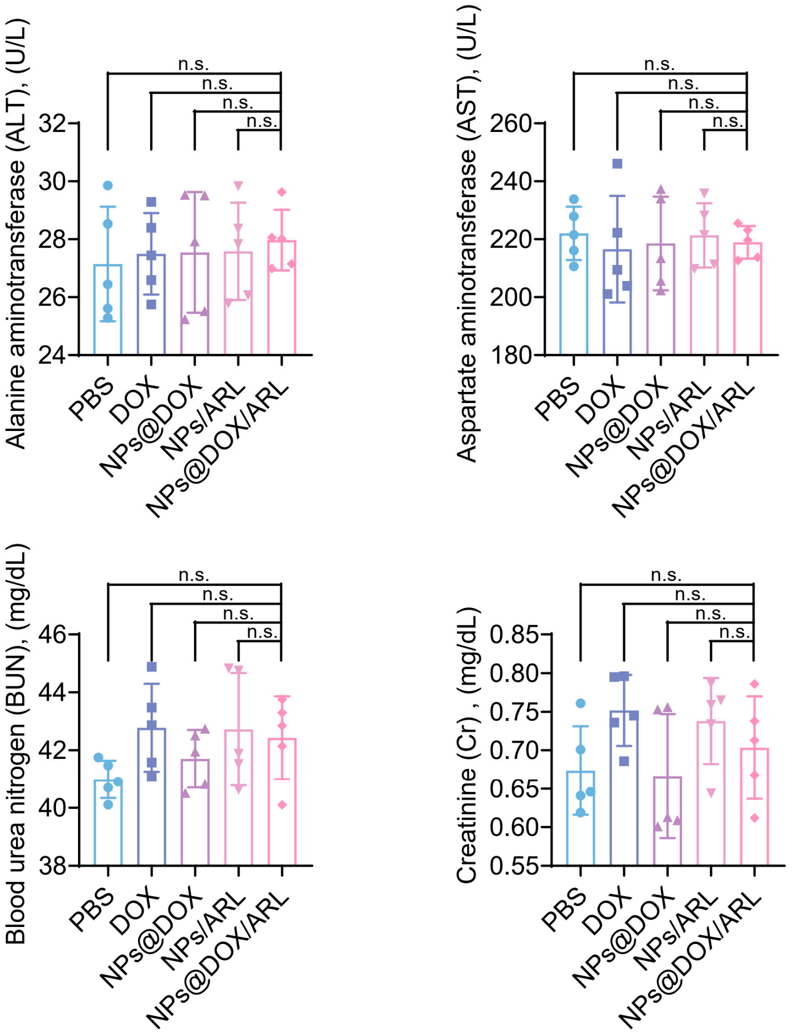

Figure S9. B16 tumor-bearing mice were intravenously injected with PBS, free DOX, NPs@DOX, NPs/ARL, or NPs@DOX/ARL (DOX: 5 mg/kg; ARL67156: 2 mg/kg per injection) on days 0, 3, and 6. On day 7, Complete blood counts were performed on blood samples collected at the end of the treatment period. ALT, AST, BUN, and Cr were evaluated.

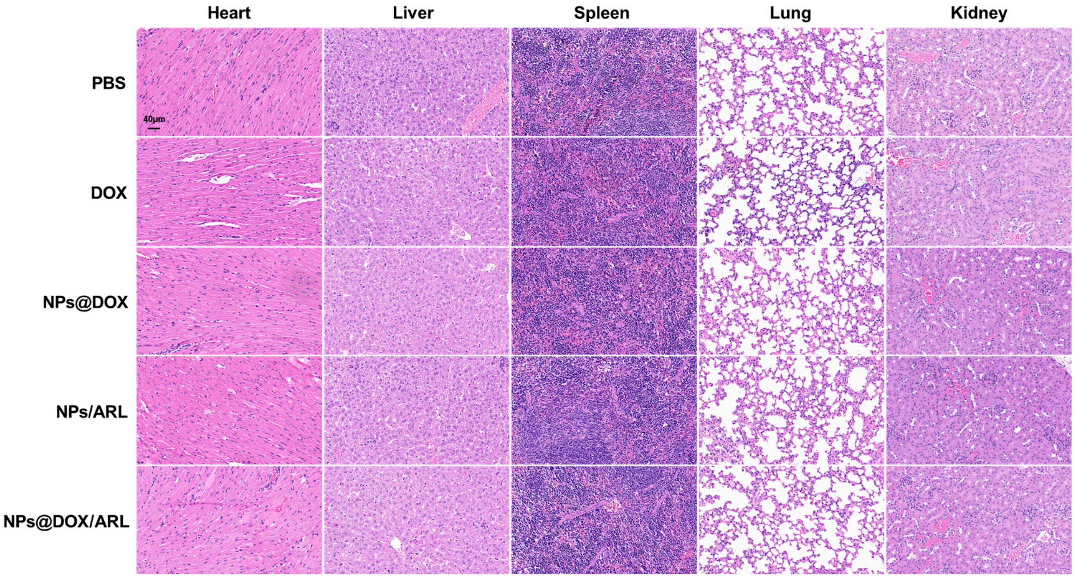

Figure S10. H&E staining of major organs.
